# Effect of an Air Stream Directed Across the Tooth on the Degree of Conversion and Temperature of Preheated Bulk-Fill Resin-Based Composites

**DOI:** 10.3390/ma19143107

**Published:** 2026-07-20

**Authors:** Cristiane Maucoski, Juliana Anany Gonzales Guarneri, Maria Tereza Hordones Ribeiro, Milena Ferreira Machado, Vinicius Borges Oliveira, Richard Bengt Price, Cesar Augusto Galvão Arrais

**Affiliations:** 1 Department of Dental Clinical Sciences, Faculty of Dentistry, Dalhousie University, Halifax, NS B3H 4R2, Canada; cmaucoski@dal.ca (C.M.); juliana.guarneri@dal.ca (J.A.G.G.); 2 Department of Restorative Dentistry, State University of Ponta Grossa, Ponta Grossa 84030-900, PR, Brazil; ferreiramachado.milena@gmail.com (M.F.M.); vinicius_borges0107@outlook.com (V.B.O.); cesararrais@yahoo.com.br (C.A.G.A.); 3 Department of Operative Dentistry and Dental Materials, School of Dentistry, Federal University of Uberlândia, Uberlândia 38405-320, MG, Brazil; mthribeiro.tete@gmail.com

**Keywords:** heated resin-based composite, degree of conversion, intrapulpal temperature, dental curing lights, air-cooling

## Abstract

Directing a stream of air across the tooth when light curing resin-based composites (RBCs) may affect their degree of conversion (DC), maximum rate of polymerization (RP_max_), and in vitro intrapulpal temperature. **Methods**: Filtek One Bulk Fill and VisCalor bulk were preheated according to the manufacturer’s instructions and used to fill a Class I cavity in a molar at 32 °C. A stream of air at either 15 or 30 psi was directed across the tooth from an air-water syringe positioned 1 cm from the buccal surface. The RBCs were light-cured for 20 s using the Bluephase N, and the DC and RP_max_ were determined from real-time FT-IR data. A T-type thermocouple positioned inside the pulp chamber recorded the temperature. DC and RP_max_ data were analyzed using one-way ANOVA, whereas temperature was analyzed using two-way ANOVA, followed by Tukey post hoc tests. **Results**: Directing a stream of air at the tooth produced no significant differences in the DC and RP_max_. The temperature change (ΔT) decreased compared to when no air was delivered. No significant difference in ΔT was found between the two air pressures. **Conclusions**: Directing a stream of air across the tooth when the preheated RBC was inserted did not affect the polymerization kinetics. The air stream reduced the temperature rise inside the pulp chamber as the RBC was light-cured. **Clinical Significance**: Directing a stream of air across the tooth at either 15 psi or 30 psi during light curing is an easy method to reduce the temperature increase inside the pulp chamber without affecting the polymerization kinetics of the evaluated preheated RBCs in this in vitro model.

## 1. Introduction

The energy delivered from high-power light-curing units (LCUs) may lead to an excessive temperature increase in the soft tissues [1,2] and inside the pulp [3,4,5,6,7]. However, the temperature rise will depend on the amount of energy delivered and the exposure time [7]. It is well-established that irradiance levels exceeding 1200 mW/cm^2^ for 10 s or more can lead to a temperature increase that may damage both soft tissues and the pulp if the remaining dentin is thin [8]. When light curing a resin-based composite (RBC), the magnitude of the temperature increase is related to the emission spectrum, light output mode, light guide tip, power, irradiance, exposure time, and the energy delivered [9,10]. In addition, the RBC used influences the temperature increase [11,12,13]. Therefore, the combined effect of the exothermic reaction from the RBC and the light from the LCU can produce a temperature increase greater than 5.5 °C at the bottom of the RBC restoration [9], potentially causing irreversible pulp damage [14].

The real-time polymerization kinetics and degree of conversion (DC) of the RBC can be measured with micro-Raman or Fourier Transform Infrared (FT-IR) spectroscopy [15,16]. The DC depends on the RBC, emission spectrum, irradiance, power, exposure time, radiant exposure, temperature and volume of the RBC [16,17,18]. Although most studies measure DC at room temperature, this does not represent intraoral or pulpal temperatures after cavity preparation [3]. This difference is relevant in the study of reaction kinetics, because temperature affects the reaction rate and the final DC [19,20,21,22]. According to the Arrhenius equation, the reaction rate increases exponentially with temperature; in many systems a 10 °C rise can double the reaction rate and a 10 °C difference is commonly observed between the temperature on the laboratory bench and the temperature inside the tooth [23]. When the RBC is at a higher temperature, it can improve the handling and the adaptation of the RBC to the walls of the cavity [19,24,25,26]. For these reasons, heating the RBC before insertion into the cavity has become popular among clinicians. However, the temperature of the heated RBC falls rapidly once the RBC is inserted into the tooth. Thus, the increases in the DC that have been reported in some laboratory studies when using heated RBCs are unlikely to occur clinically [27,28].

A variety of commercially available devices are designed to preheat RBCs, with heating temperatures as high as 68 °C [24]. Consequently, the impact of the temperature rise associated with heated RBCs has become a concern. Strategies capable of reducing temperature rise while maintaining adequate polymerization are clinically relevant, as they may improve the biological safety of restorative procedures without compromising material performance [13]. Some approaches have been proposed to avoid a dangerous increase in pulpal temperature (PT), such as reducing the exposure time from the LCU [8] or by directing a stream of air across the tooth during light exposure [4,29,30]. Directing a stream of air across the tooth is known to reduce its temperature [29], thereby increasing the amount of thermal energy required to raise the temperature of the tooth structure [4]. Although the stream of air can prevent the PT from increasing during light curing [4,30], it may also reduce the DC of the RBC. A previous study evaluated the effect of an air stream on in vitro intrapulpal temperature changes and hardness values, demonstrating that air pressures of 15 and 30 pounds per square inch (psi) reduced PT without adversely affecting hardness values [31]. However, to date, no studies have investigated the effect of directing a stream of air across the tooth on the DC values of heated bulk-fill RBCs. Therefore, this study evaluated the DC of heated bulk-fill RBCs, the maximum rate of polymerization (RP_max_), and the in vitro pulpal temperature when a stream of air was simultaneously applied with light exposure from a multi-peak light-emitting diode (LED) LCU. The null hypotheses are:

(1) Directing a stream of air across the tooth when light curing preheated bulk-fill RBCs will not affect the DC and RP_max_ values at the bottom of the heated bulk-fill RBCs when compared to when no air is used;
(2) Reducing the air pressure from 30 to 15 pounds per square inch (psi) will not affect the DC and RP_max_ values at the bottom of the preheated bulk-fill RBCs;
(3) Directing a stream of air across the tooth when light curing preheated bulk-fill RBCs will not affect the intrapulpal temperature.

## 2. Materials and Methods

### 2.1. Analysis of the Light Emitted by the LED LCU

One multi-peak LED LCU, the Bluephase N (Ivoclar, Schaan, Liechtenstein), with a 9 mm tip diameter, was used in its high-output mode. The power (mW), radiant exitance or tip irradiance (mW/cm^2^), and radiant exposure (J/cm^2^) emitted in the wavelength range between 350 and 550 nm were recorded using a fiber-optic spectroradiometer (Flame-T; Ocean Optics, Orlando, FL, USA) connected to a 6-inch diameter integrating sphere (Labsphere, North Sutton, NH, USA) that had been previously calibrated using an internal NIST-referenced calibration lamp (ICS-600; Labsphere, North Sutton, NH, USA). The LCU tip was positioned at the entrance of the sphere to capture all the light emitted from the LCU at a distance of 0 mm. The data was recorded using OceanView software 2.0 (Ocean Optics, Orlando, FL, USA), which provided the spectral radiant power and total emitted power. Power output was measured in triplicate. The internal optical tip diameter of the LCU tip was measured using a digital caliper (Mitutoyo, Mississauga, ON, Canada), and the optical emission area of each light-curing tip was calculated. The power (mW) was then divided by the area of the tip to obtain an averaged radiant exitance (mW/cm^2^) across the light tip. This irradiance was multiplied by the exposure time to provide the radiant exposure (J/cm^2^) delivered across the light tip.

### 2.2. Temperature Analysis of the Preheated RBCs

Two bulk-fill RBCs were used: Filtek One Bulk Fill (FOB; Solventum, St. Paul, MN, USA) and VisCalor bulk (VIS; VOCO, Cuxhaven, Germany). The manufacturers’ information, lot number, and shade are reported in Table 1. Before inserting into the cavity, the RBCs were heated in the Caps Warmer (VOCO, Cuxhaven, Germany) with the VisCalor Dispenser (VOCO, Cuxhaven, Germany) or in the Calset composite warmer (AdDent Inc., Danbury, CT, USA) with the CoMax Dispenser (AdDent, Danbury, CT, USA) to a target temperature of 68 °C for 5 min for FOB and 3 min for VIS, both according to the manufacturer’s recommendations [32,33]. The temperature of the RBCs was recorded using a T-type thermocouple connected to a temperature device (Physitemp Thermes; Physitemp Instruments, Clifton, NJ, USA) inserted into the RBC to measure the peak temperature and the temperature drop of the RBC after 20 s, simulating clinical placement time. Peak temperature was defined as the highest temperature recorded every 0.2 s by the thermocouple immediately after heating in the heating device. Using software (DASYLab 11; Physitemp Instruments, Clifton, NJ, USA), the temperature was recorded for 20 s while it was exposed to room air, in accordance with the manufacturer’s suggested time allowed for applying the heated RBC into the cavity [33].

### 2.3. Degree of Conversion (DC) and Polymerization Kinetics

**Figure 1:**
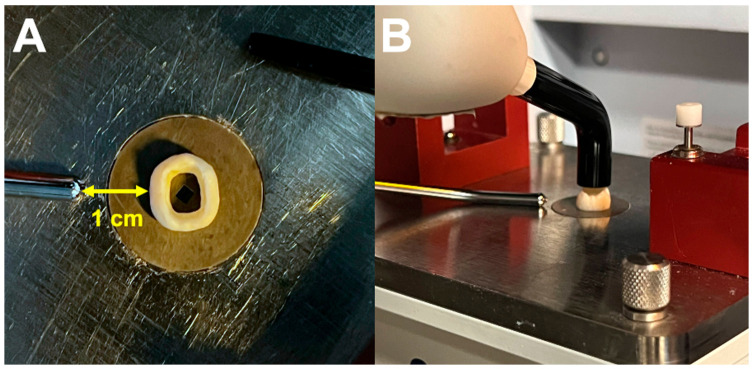
(**A**): Tooth with the divergent Class I cavity placed over the detector on the ATR unit. The tip of an air-water syringe was placed 1 cm from the buccal surface of the tooth, and the air stream was directed at a 90° angle relative to the tooth surface. (**B**): The LCU tip was centered over the tooth, with the light tip touching the cusps.

This study was approved by the University Ethics Committee (#5.427.825) and two molar teeth were obtained from the University Tooth Bank, which were stored according to the institutional protocol for extracted teeth until use. For the polymerization kinetics measurements, a divergent Class I preparation was made in one extracted intact lower third molar. The cavity was 4 mm deep, 8 mm wide and 7 mm long. The cervical portion of the tooth was ground away until the pulpal floor was removed. This preparation allowed direct contact between the bottom surface of the RBC and the ATR diamond crystal to provide real-time measurements of polymerization kinetics at the deepest portion of the restoration. The tooth was placed over the diamond prism (Figure 1) of an Attenuated Total Reflectance (ATR crystal) unit (Golden Gate; Specac, Orpington, Kent, UK) connected to an FT-IR spectrometer (Vertex 70; Bruker, Billerica, MA, USA) [34]. To simulate the temperature at the pulpal floor near the pulp horn during a deep cavity preparation, the temperature of the ATR stage was set to 32 °C [3,25]. Before inserting into the cavity, a hydrophilic gel (K-Med; CIMED, Pouso Alegre, MG, Brazil) was applied to the preparation walls as a lubricant, and the RBCs were preheated in the Calset composite warmer (AdDent) with the CoMax Dispenser (AdDent) to the same target temperature (68 °C) used in the temperature characterization and intrapulpal temperature experiments. Therefore, the actual composite temperature at placement, rather than the warming device itself, was considered the critical experimental parameter. With the tooth placed over the ATR detector, the RBCs were inserted in one increment according to the manufacturer’s instructions. The order of the experimental conditions was randomized prior to testing. The LCU tip was positioned perpendicularly and centered on the occlusal surface, touching the cusp tips. The filling procedure took approximately 20 s, which was the same time that was used to measure the temperature of the RBC exposed to room air. The tip of a standard air-water syringe was placed 1 cm away from the buccal surface (Figure 1), and the real-time FT-IR readings started after placing the RBC into the tooth. Starting 3 s before the light was turned on, a steady stream of dry air was directed towards the tooth during the light exposure [4]. Air stream pressures of 15 psi and 30 psi [31], representative of those commonly used in dental offices, were evaluated, and an external pressure regulator was used to standardize the syringe pressure. The control group received the same treatment but did not receive any air stream directed across the tooth. The RBC was light-cured according to the RBC manufacturer’s recommendations for 20 s in the high-output mode. For each DC measurement, the first 50 scans of the uncured RBCs were collected between 750 and 1900 cm^−1^ at a resolution of 8 cm^−1^. Following standard methods [35], fifty scans were averaged at 135 s after the start of light curing to determine the immediate DC. This immediate DC was calculated from the changes in the ratios of the aliphatic-to-aromatic C=C absorption peaks (1635 cm^−1^/1609 cm^−1^) between the uncured and cured states, as determined from the infrared spectra. The maximum conversion rate, RP_max,_ corresponded to the highest polymerization rate (percentage) at 1 s intervals and was calculated from the differences in DC values measured throughout each specimen’s DC recordings. Ten measurements were performed per group condition. Between measurements, the RBC was removed, and the cavity walls were visually inspected and gently cleaned to remove any residual composite material or debris. Care was taken to avoid scratching the cavity walls or altering the cavity dimensions throughout the experimental procedure.

### 2.4. In Vitro Intrapulpal Temperature Measurements

**Figure 2:**
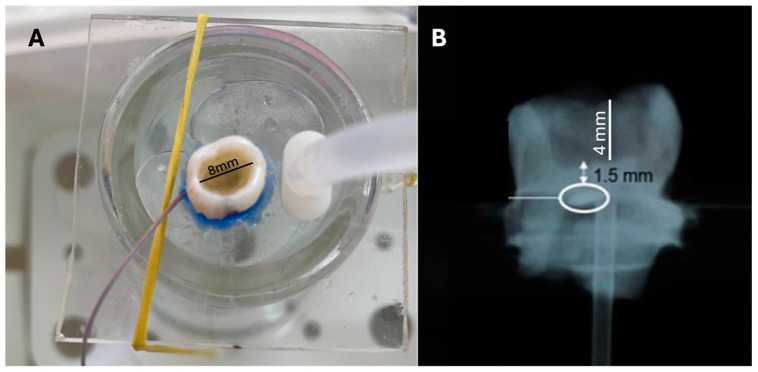
(**A**): Tooth with the divergent Class I cavity positioned in the Kitasato bottle. The cavity dimensions were 8 mm wide and 7 mm long. (**B**): Radiograph of the position of the thermocouple. The remaining dentin thickness was 1.5 mm, and the cavity was 4 mm deep. The white circle represents the position of the thermocouple inside the pulp chamber.

To estimate intrapulpal temperature during light curing of the RBC, with and without an air stream directed across the tooth, a second extracted intact mandibular third molar was used. This tooth had a Class I cavity preparation with the same dimensions as the tooth used for the polymerization-kinetics analysis (Figure 2). The same light-curing protocol and experiment groups were used. The roots of the tooth were sectioned 4 mm below the cement-enamel junction, and the root canals were cleaned and enlarged using Gates-Glidden burs (Dentsply Sirona, Charlotte, NC, USA) at low speed. A small access was created on the proximal surface using a high-speed diamond bur (#1011; KG Sorensen, Cotia, SP, Brazil) under constant irrigation, next to the cement-enamel junction, to allow insertion of a T-type thermocouple inside the pulp chamber. To fix the position of the thermocouple in the pulp chamber throughout all testing, the thermocouple was sealed in place using a flowable RBC (Opallis Flow; FGM, Joinville, SC, Brazil). A radiograph was obtained to confirm the thermocouple’s position within the pulp chamber (Figure 2). The tooth was placed through an opening made through the center of an acrylic plate, and the tooth/plate gap was sealed using the same flowable RBC. The end of a plastic tube was connected to one root, and the other end was connected to an infusion pump (RS700 RZ, São Paulo, SP, Brazil) set to deliver water at a simulated pulpal flow rate. Recognizing that age, tooth location, inflammation, and local anesthetics can all affect flow rate, the flow rate through the pulp chamber was set at 0.008 mL/min (8 μL/min) [36].

The acrylic plate with the tooth was placed in a Kitasato bottle and immersed in a temperature-controlled water bath so that the water flow within the pulp chamber was heated to a temperature corresponding to the physiological pulpal baseline temperature of 32 °C [3]. Two other T-type thermocouples were connected to the temperature acquisition system. One was immersed in the water bath, and the other was placed in the water inside the Kitasato bottle. This ensured the system supplied the pulp chamber with the correct water temperature. The T-type thermocouple was connected to a Physitemp Thermes (Physitemp Instruments) analog-to-digital converter and monitored using DASYLab 11 software (Physitemp Instruments). This real-time temperature data collection began before the RBC was inserted into the cavity and continued throughout the restorative procedure. A hydrophilic gel was applied to the preparation walls as a lubricant, and a small piece of dental floss was left inside the preparation to aid in removing the cured RBC after it had been light-cured. No bonding agents were applied to the preparation walls. The order of the experimental conditions was randomized prior to testing. As in the polymerization kinetics analysis, all RBC restorations were performed in the same tooth to eliminate variability arising from differences in tooth anatomy and thermocouple positioning within the pulp chamber.

Before inserting the RBC into the cavity, the RBC capsule was placed in the VisCalor Dispenser (VOCO) and heated in the Caps Warmer device (VOCO) to the target temperature of 68 °C for 5 min for FOB and 3 min for VIS [32,33]. The RBCs were then inserted into the cavity in bulk following the manufacturers’ instructions. The same two pressures of air were delivered (15 psi and 30 psi), and the temperature was recorded in real-time, every 0.2 s. The tip of the air syringe was positioned 1 cm from the buccal surface, while the LCU tip was placed directly on the occlusal surface. The preparation was filled with RBC in a single increment, and the air stream was directed across the tooth starting from 3 s before light-curing until it was completed (20 s). After the baseline temperature was reestablished, the RBC was removed by pulling on the embedded dental floss, and a new test was carried out on the same tooth using the next randomly assigned condition. Between measurements, the cavity walls were inspected and gently cleaned without altering the cavity dimensions. Each condition was repeated 10 times. The peak temperature during light curing and the difference between the peak and baseline temperatures (ΔT) were obtained. All temperature measurements were performed in the same room with a room temperature of 23 ± 1 °C.

### 2.5. Statistical Analysis

The sample size was determined using G*Power [37], version 3.1.9.7 (Universität Düsseldorf, Düsseldorf, Germany), based on data from a pilot study. A one-way ANOVA design (three groups) was considered for DC and polymerization kinetics, with α = 0.05 and power = 90% (β = 0.10). Effect sizes were estimated from previous studies and a pilot dataset [36,38], resulting in f = 0.72 for DC and f = 0.81 for ΔT. The largest required sample size was adopted, and a minimum sample size of 10 experimental runs per group was used. The n = 10 measurements represented ten independently prepared restorations evaluated within the same standardized tooth model. Each experimental run consisted of a new restoration, which was inserted, light-cured, removed, and followed by cleaning of the tooth cavity before the subsequent run. The tooth served as a constant experimental substrate to minimize anatomical variability among measurements. The data distribution and homogeneity of variances were checked using the Shapiro–Wilk and Levene tests, respectively. The DC values at 135 s and the maximum rate of polymerization (RP_max_) were subjected to a one-way analysis of variance (ANOVA) test followed by Tukey post hoc tests. The temperature analysis was subjected to a two-way ANOVA followed by Tukey post hoc tests. No comparison between the RBCs was performed for DC and RP_max_. All statistical analyses were conducted using a significance level of α = 0.05.

## 3. Results

### 3.1. Analysis of the Light Emitted by the LED LCU

**Figure 3:**
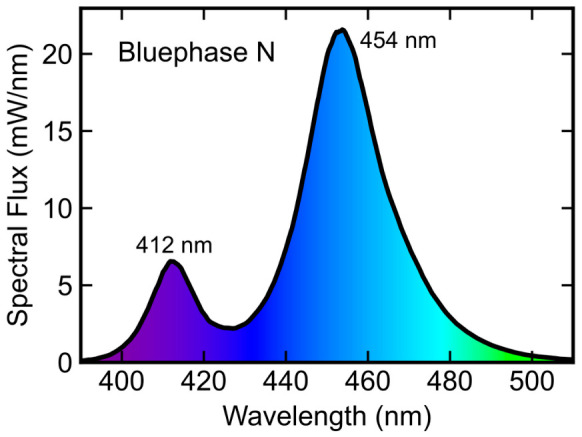
Emission spectra from the Bluephase N LCU.

Figure 3 shows the peak wavelengths (nm) and the spectral radiant power from Bluephase N in high mode. The Bluephase N is a multi-peak broadband LED LCU that delivers two wavelength peaks, one in the violet region (λ1 = 412 nm) and one in the blue region (λ2 = 454 nm). In high mode, the Bluephase N emitted 727 mW (±45.2) and an average irradiance across the tip of 1143 mW/cm^2^ (±71.1). Thus, a radiant exposure of 22.8 J/cm^2^ (±1.4) was delivered in 20 s.

### 3.2. Temperature Analysis of the Heated Composites Inside the Capsule

Table 2 reports the peak temperature of the RBCs immediately after removal from the heating device, the temperature after a 20 s period simulating clinical placement, and the corresponding temperature change (ΔT). For both RBCs, substantial cooling occurred during the 20 s period regardless of the heating device used. When heated using the Caps Warmer, the temperature decreased by 23.5 °C for FOB and 28.7 °C for VIS after 20 s. The peak temperatures were 69.1 °C (±0.0) for FOB and 67.9 °C (±0.6) for VIS, decreasing to 45.6 °C (±1.8) and 39.2 °C (±1.6), respectively. When heated using the Calset warmer, the peak temperatures were lower, reaching 65.1 °C (±0.4) for FOB and 64.2 °C (±1.0) for VIS. After 20 s, the temperatures decreased to 47.3 °C (±3.4) and 43.4 °C (±3.4), corresponding to temperature reductions of 17.8 °C and 20.8 °C for FOB and VIS, respectively.

### 3.3. Degree of Conversion (DC) and Polymerization Kinetics

The DC values for VIS and FOB for the different air stream conditions are reported in Table 3. No significant differences were found in DC and RP_max_ values across conditions. Directing a stream of air across the tooth at either 15 or 30 psi did not significantly affect the DC or RP_max_ values of either RBC (*p* > 0.05). For FOB, the DC values ranged from 51.8 ± 3.6% to 52.2 ± 2.0%, while RP_max_ ranged from 3.5 ± 0.2%/s to 3.6 ± 0.5%/s. For VIS, the DC values ranged from 35.7 ± 0.6% to 36.0 ± 0.8%, and RP_max_ ranged from 3.9 ± 0.6%/s to 4.2 ± 0.5%/s. Tukey post hoc analysis confirmed that no significant differences existed among the air-cooling conditions within the same material (*p* > 0.05).

**Figure 4:**
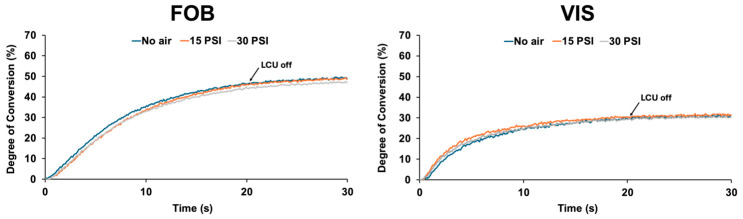
Representative 30 s degree of conversion of Filtek One Bulk Fill (FOB) and VisCalor bulk (VIS) when they were light-cured using different conditions, showing that directing a stream of air at 15 psi or 30 psi across the tooth did not affect the degree of conversion under the conditions tested.

Figure 4 shows the representative polymerization kinetics curves for FOB and VIS when they were light-cured concomitantly under three conditions (no air stream, air stream directed across the tooth at a pressure of 15 psi, and at 30 psi). For both RBCs, the DC increased rapidly during the 20 s light exposure period and continued to increase at a slower rate after the LCU was switched off. The conversion profiles obtained with no air stream, 15 psi, and 30 psi were similar throughout the recording period.

### 3.4. In Vitro Pulpal Temperature Analysis

Table 4 reports the mean (SD) baseline, peak and temperature change (ΔT) when the RBCs were light-cured. A two-way ANOVA demonstrated significant effects of RBC and air stream condition on both peak temperature and ΔT (*p* < 0.001), while no significant interaction between factors was observed (*p* > 0.05). For both outcomes, the no-air condition resulted in significantly higher values than either 15 psi or 30 psi air stream application (*p* < 0.001), whereas no significant difference was found between the two air stream pressures (*p* > 0.05).

The mean temperature change was 5.4 ± 0.3 °C for FOB when no air stream was directed over the tooth. The ΔT decreased when a stream of air was delivered across the tooth (2.2 ± 0.3 °C for 15 psi and 2.1 ± 0.2 °C for 30 psi). The same pattern occurred with VIS (4.9 ± 0.2 °C when no air was used, 2.1 ± 0.2 °C for 15 psi and 1.8 ± 0.2 °C for 30 psi). There was a statistically significant difference between the groups when no air stream was directed across the tooth compared to when a stream of air was directed across the tooth. No statistically significant difference was detected in the effects of the two air pressures (15 psi or 30 psi) on the temperature (*p* = 0.098 for peak temperature and *p* = 0.108 for ΔT). There was a statistically significant difference between the two RBCs regarding intrapulpal temperature for both peak temperature and ΔT (*p* = 0.001 and *p* < 0.001, respectively). FOB showed overall higher temperature values than VIS (Table 4).

## 4. Discussion

It has been reported that directing an air stream across the tooth can prevent an unacceptable rise in temperature during light curing of RBCs [4,29,30]. In a previous in vivo study, the authors reported that directing a stream of air at 28 psi prevented an unacceptable temperature increase [4]. For this reason, directing a stream of air across the tooth has been recommended [4,30,31]. One concern regarding the use of an air stream during polymerization is that the resulting cooling effect could adversely affect the mechanical properties of RBCs. Recent evidence indicates that the application of an air stream at air pressures of 15 and 30 psi significantly reduces temperature rise while maintaining Vickers hardness values [31]. The present study evaluated the polymerization kinetics of heated bulk-fill RBCs and the in vitro pulpal temperature when a stream of air was applied during light exposure. Under the conditions tested, no differences were found in DC and RP_max_ values between RBCs exposed to a stream of air and the control group that received no air across the tooth (Table 3). Thus, the hypothesis that directing a stream of air across the tooth as the RBC is light-cured will not affect the DC and RP_max_ values at the bottom of the heated bulk-fill RBCs when compared to the values from the control group was accepted. Since a stream of air will cool the enamel and dentin structures [4], the temperature gradient in the surrounding structures is greater, leading to an increased heat loss from the RBC. For this reason, the current findings were not expected. The temperature values of the RBCs in the heaters ranged from approximately 64 °C to 69 °C (Table 2), and these values were close to those claimed by the manufacturers and those reported in the literature [24]. The dentin that had been cooled by the stream of air should absorb the heat from the RBC, causing a drop in the temperature of the RBC. Although different warming devices were used during the DC and intrapulpal temperature measurements, the warming device itself was not considered an experimental variable. However, it should be noted that before the RBC was inserted into the cavity preparation, a significant temperature drop had already occurred (Table 2). This agrees with previous findings [25,39]. In addition, because the RBC temperature was still higher than the temperature of the pulpal floor of the cavity (32 °C), the thermal energy from the heated composite dissipated from the RBC toward the dentin and enamel [40]. This resulted in a further temperature decrease within the RBC before the stream of air was directed over the tooth. Since the temperature affects the molecular mobility within the RBC [21,24], a lower monomer conversion would be expected compared to the values obtained when the RBC was close to 68 °C [22]. No comparison was made between materials for DC and polymerization kinetics. The lower DC values observed for VisCalor bulk compared with Filtek One Bulk Fill should not be interpreted as an effect of the air-cooling procedure. These materials differ in composition and polymerization behavior, all of which can influence the final degree of conversion.

The effect of the air stream on DC and RP_max_ was not statistically detectable regardless of the two different air pressures used (Table 3, Figure 4). Therefore, the second research hypothesis was accepted. The RP_max_ was approximately 4%/s at the bottom of 4 mm of the RBC. Interestingly, directing a stream of air across the tooth did not significantly affect the RP_max_ (Table 3). A previous investigation reported an increase in RP_max_ when the composite was heated, reaching 9.8%/s at the bottom of a 2 mm deep cavity [21]. Another study reported that increasing the temperature of the RBC to 35 °C increased the RP_max_ to 12%/s [41]. The differences between these RP_max_ values are most likely due to differences in the methods used, or the RBC, since the RP_max_ and DC can be different depending on which RBC is used [42].

Heated RBCs have been used in direct and indirect restorations [43,44,45,46]. For indirect restorations, the RBC generally returns to room temperature by the time it is placed in the cavity, as shown in the present study, where the temperature dropped rapidly within the first 20 s (Table 2). However, in direct restorations, the RBC will still be warm after heating, potentially leading to a higher temperature rise upon insertion. Although heating the RBC before inserting it into the cavity has some benefits, a temperature increase in the pulp chamber above 5.5 °C may lead to irreversible damage to the pulp tissue [14]. In one in vivo study, the authors found that the temperature at the pulpal floor increased by approximately 6 °C when the cavity was filled with heated RBC compared to a room temperature RBC [25]. A different study reported that using heated composite (initially at 54 °C or 60 °C) was safe. This study found that the temperature of the RBC fell rapidly after it was removed from the heater, and the significant factor contributing to the rise in pulpal temperature was the effect of the LCU, not the RBC [20]. When analyzing the benefits of directing a stream of air across the tooth to control the pulp temperature rise, the results from this study showed that directing a stream of air across the tooth resulted in a lower temperature increase for both RBCs tested (Table 4) and that the pressure of the air stream did not have an effect on the temperature increase (Table 4). Therefore, the third hypothesis that directing a stream of air across the tooth filled with preheated bulk-fill RBCs will not affect the increase in the intrapulpal temperature during light curing was rejected.

Even though the 5.5 °C threshold is often used as a reference for pulp temperature increase, studies showing that transient intrapulpal temperature increases of 8.9–14.7 °C found no histological evidence of pulp damage [47]. Therefore, while the threshold is important to standardization, its direct clinical applicability is debatable, as the duration of temperature increase [48], remaining dentin thickness [5], and the presence of pulpal blood flow [49] all influence the biological response to heat. Besides the effect of using preheated RBCs, there is a risk of pulpal damage when using powerful LCUs for a prolonged time [8]. The heated air around the irradiated tooth and RBC causes some of the temperature increase during light curing [29], along with the thermal behavior of the enamel and dentin when the tooth is exposed to light from the LCU [40]. In the present study, although directing a stream of air across the tooth was expected to decrease the DC, changing the flow of air from 15 psi to 30 psi across the tooth did not influence the polymerization kinetics at the bottom of FOB and VIS (Table 3), but it did result in a lower temperature increase inside the pulp compared to the control group (Table 4). For this reason, these findings suggest that directing a stream of air across the tooth is beneficial for controlling the temperature increase without adversely affecting polymerization kinetics (Table 3). This is generally consistent with a previous in vitro Class V study [31], although in the present study no statistically significant difference was detected between 30 psi and 15 psi (Table 4). In addition, care should be taken to ensure that only air is delivered and that water is not inadvertently activated. The clinical implementation of this technique may also require a dental assistant or a four-handed working approach. Pressures outside the 15–30 psi range evaluated in this study may produce different thermal outcomes. Therefore, future studies should investigate whether other air pressures produce different thermal responses.

As the thickness of the remaining dentin [5] and overall tooth dimensions are major determinants of intrapulpal temperature, using multiple teeth would have introduced significant confounding variability. In addition, the thermocouple’s position would change in a multiple-teeth model, potentially influencing the temperature results. Thus, the use of a standardized tooth allowed a high degree of standardization (geometry, cavity dimensions, and positioning) and ensured that measured differences were attributable solely to the RBC and cooling technique used. It is important to note that following each measurement cycle, the polymerized composite was removed entirely, resetting the system to an identical baseline for the next test. Therefore, each experimental run was treated as an independent observation of the material-system interaction [36,50,51,52]. While this design optimizes internal precision, the generalizability of the absolute temperature values to teeth of different morphologies is a recognized limitation. Consequently, the present design does not permit estimation of between-tooth variability, and the findings should be interpreted as applying to this highly controlled in vitro setup.

Only two air pressures, two types of bulk-fill RBCs, and two lower molar teeth were used in this in vitro study. Therefore, the results should be interpreted within the context of this highly standardized in vitro model. The findings may differ when other RBCs are used, air pressures are applied, larger restoration volumes are used, or different teeth are considered. In particular, the use of a limited number of teeth with repeated measurements does not account for variations in tooth morphology, dentin thickness, or cavity geometry, which may influence heat transmission toward the pulp [8]. Since the present study measured the intrapulpal temperature rise, the temperature of the RBC itself during curing was not directly measured. Therefore, our findings should be interpreted as reflecting the effects of preheating rather than the effects of curing the RBC at a temperature of 68 °C. Moreover, different pulp fluid flows [52] and physiological heat dissipation in an in vitro model may result in intrapulpal temperature changes that are different from those observed in the mouth. Further investigations incorporating different tooth substrates and clinically relevant conditions, as well as the evaluation of additional mechanical properties, such as hardness, are warranted to better understand the effects of directing an air stream during light curing.

## 5. Conclusions

Within the limitations of this in vitro study, it was concluded that:

(1) Directing a stream of air across the tooth at either 15 psi or 30 psi, starting after the RBC was inserted, did not influence the polymerization kinetics of RBCs that had been preheated to a target temperature of 68 °C.
(2) Directing a stream of air across the tooth reduced the temperature increase inside the pulp chamber as the RBCs were light-cured.

## Figures and Tables

**Table 1:** Manufacturer information of the bulk-fill resin-based composites used in the study.

RBC		Lot Number	Manufacturer	Shade	Recommended Minimum Irradiance, Wavelength Range and Exposure Time
Filtek One Bulk Fill	FOB	NF16925 NC44145	Solventum, St. Paul, MN, USA	A2	1000 mW/cm^2^ 400–500 nm for 20 s
VisCalor bulk	VIS	2106342	VOCO, Cuxhaven, Germany	A2	1000 mW/cm^2^ 400–500 nm for 20 s

**Table 2:** Mean (±standard deviation) peak temperature (°C), temperature after a 20 s period simulating clinical placement, and temperature change (ΔT) between the mean values of the resin-based composites.

RBC	Heater	Peak Temperature	RBC Temperature After 20 s	ΔT
FOB	Caps Warmer	69.1 (±0.0)	45.6 (±1.8)	23.5
	Calset	65.1 (±0.4)	47.3 (±3.4)	17.8
VIS	Caps Warmer	67.9 (±0.6)	39.2 (±1.6)	28.7
	Calset	64.2 (±1.0)	43.4 (±3.4)	20.8

**Table 3:** Mean (±standard deviation) degree of conversion (%) and RP_max_ (±standard deviation) of Filtek One Bulk Fill and VisCalor bulk with and without the effect of an air stream directed across the tooth.

	Filtek One Bulk Fill (FOB)		VisCalor Bulk (VIS)	
	Degree of Conversion (%)	RP_max_ (%/s)	Degree of Conversion (%)	RP_max_ (%/s)
No air stream	51.8 (±3.6) A	3.5 (±0.4) a	36.0 (±0.8) A	4.2 (±0.5) a
15 psi	52.0 (±7.2) A	3.6 (±0.5) a	35.8 (±1.1) A	4.0 (±0.3) a
30 psi	52.2 (±2.0) A	3.5 (±0.2) a	35.7 (±0.6) A	3.9 (±0.6) a

Uppercase letters compare degree of conversion values within the same material; lowercase letters compare RP_max_ values within the same material. Means followed by similar letters are not significantly different (Tukey post hoc test, *p* > 0.05). No statistical comparisons were made between RBCs.

**Table 4:** Mean (±standard deviation) baseline temperature, peak temperature and the temperature change (ΔT) of Filtek One Bulk Fill and VisCalor bulk when light-cured for 20 s with and without a 15 or 30 psi air stream across the tooth.

	Filtek One Bulk Fill (FOB)			VisCalor Bulk (VIS)		
	Baseline °C	Peak Temperature °C	ΔT °C	Baseline °C	Peak Temperature °C	ΔT °C
No air stream	32.5 (±0.5)	37.9 (±0.5) Aa	5.4 (±0.3) Aa	32.4 (±0.2)	37.4 (±0.2) Ab	4.9 (±0.2) Ab
15 psi	32.5 (±0.4)	34.7 (±0.4) Ba	2.2 (±0.3) Ba	32.5 (±0.3)	34.6 (±0.2) Bb	2.1 (±0.2) Bb
30 psi	32.4 (±0.4)	34.5 (±0.3) Ba	2.1 (±0.2) Ba	32.5 (±0.4)	34.3 (±0.3) Bb	1.8 (±0.2) Bb

Uppercase letters compare air stream conditions within the same RBC. Lowercase letters compare RBCs within the same air stream condition and variable. Means followed by similar letters are not significantly different (Tukey post hoc test, *p* > 0.05).

## Data Availability

The original contributions presented in this study are included in the article. Further inquiries can be directed to the corresponding author.
